# Geographical Barriers Impeded the Spread of a Parasitic Chromosome

**DOI:** 10.1371/journal.pone.0131277

**Published:** 2015-06-25

**Authors:** María Inmaculada Manrique-Poyato, María Dolores López-León, Josefa Cabrero, Ricardo Gómez, Francisco Perfectti, Juan Pedro M. Camacho

**Affiliations:** 1 Departamento de Genética, Facultad de Ciencias, Universidad de Granada, 18071, Granada, Spain; 2 Departamento de Ciencia y Tecnología Agroforestal, E.T.S. de Ingenieros Agrónomos, Universidad de Castilla La Mancha, 02071, Albacete, Spain; Leibniz-Institute of Plant Genetics and Crop Plant Research (IPK), GERMANY

## Abstract

Parasitic supernumerary (B) chromosomes show high capability to spread across populations. But the existence of abrupt discontinuities in their distribution demands an explanation. The grasshopper *Eyprepocnemis plorans plorans* harbour supernumerary chromosomes in all natural populations hitherto analyzed from the Circum-Mediterranean region, with the single exception of the headwaters of the Iberian Segura River and several of its tributaries. To ascertain the causes of this distribution pattern, we analyze here the genetic structure of five natural populations collected in this zone (two +B and three -B), by means of ISSR markers. We found significant population structure, with two kinds of populations coinciding with +B and -B ones, separated by strong barriers to gene flow. This gives strong support to the hypothesis that the non-B populations precede B origin, and that B-carrying individuals from coastal zones have been able to colonize upstream areas, until geographical barriers (usually narrow canyons and arid areas surrounding them) impeded their advance.

## Introduction

Gene flow is a main determinant of population structure because different populations may evolve as a unit if gene flow is high but they may accumulate differences when gene flow is low. The amount of gene flow impeding population differentiation depends on its balance with other forces, mainly genetic drift and selection [1]. However, gene flow may be conditioned by the existence of geographical barriers limiting individual dispersion among populations in a higher extent than expected from only geographical distance. In the absence of major barriers, genetic differentiation among populations is expected to be proportional to geographical distance, so that significant isolation by distance (IBD) is apparent. But abrupt geographical barriers to gene flow may break this association causing absence of IBD despite significant genetic differentiation among populations.

The grasshopper *Eyprepocnemis plorans* subsp. *plorans* lives in humid places next to the coast and in the vicinity of river shores and adjacent irrigated cultivation areas, in a Circum Mediterranean distribution. Farming activities have no doubt served as means for this grasshopper dispersion in the last few thousand years. In natural populations from an irrigated area close to the Segura River at Murcia (Spain), downstream to the populations analysed here, it has been reported that this species displays a single generation per year, from July to March, being abundant in all types of cultivations and plant associations, due to its polyphagous character, a low exigent diet and high dispersal ability, allowing it to find new appropriate biotic conditions [2]. In addition, this species shows high reproductive output since females can yield more than 100 fertilized eggs after a single mating, although some females may yield up to 313 eggs [3]. This high reproductive potential, ubiquitous feeding and dispersal capability make *E*. *plorans* an excellent colonizer, since a single gravid female may easily found a new population. Bearing also in mind that egg productivity increases significantly with the number of matings [4], the high reproductive output of this grasshopper species explains how a coastal species of African origin [5] has reached high altitude localities such as Calasparra (385m), Mundo (520m), Caravaca (575m), Claras (640m) and Socovos (730m) in the Iberian Peninsula.

This grasshopper harbours a complex system of B chromosomes in terms of both variation and geographical range, since more than 50 different B-variants have been described in the Iberian Peninsula [6, 7] and they are present in almost all natural populations where their presence has been tested: Italy [8], Morocco [9], Dagestan [10], Balearic Islands [11], Greece [12], Armenia and Turkey [13].

In the Iberian Peninsula, B-lacking populations have only been found in the headwaters of several rivers belonging to the Segura river basin, including the Segura River itself and several of its tributaries (e.g. Mundo, Benamor and Taibilla) [14]. Since in each of these rivers the border between B presence and absence coincided with the existence of a geographical barrier, e.g. narrow steep canyons (in some of which artificial reservoirs have been built), these authors hypothesized that B chromosomes arose in coastal populations and have not yet reached the populations in the headwaters due to these geographical barriers.

The widespread presence of B chromosomes in *E*. *p*. *plorans*, reaching almost the entire geographical range of this subspecies, has been facilitated by the existence of a transmissional advantage (drive) during female meiosis [15], a property being fundamental for B chromosome invasion of new populations [16]. Gene flow is a second feature facilitating B chromosome spread, and it is actually high in *E*. *plorans* [17] thus helping to explain how these B chromosomes have reached so widespread geographical distribution despite being of recent origin [18].

In this paper we test the hypothesis that B chromosomes are absent from the headwaters area of the Segura River basin (Iberian Peninsula) because geographical barriers have impeded their advance from coastal populations [14]. For this purpose, we analysed Inter Simple Sequence Repeat (ISSR) markers in five populations, two of them carrying B chromosomes and the three remaining lacking them. These molecular markers provided estimates of historical gene flow among populations, and the analysis of population structure revealed the existence of significant barriers to gene flow which spatially coincided with both the presence of geographical barriers making it difficult this grasshopper dispersion and the distribution of B-carrying and B-lacking populations.

## Results

B chromosomes were found in two populations (Mundo and Calasparra) but not in the remaining three (Claras, Socovos and Caravaca) (S5 Table). The six primers employed yielded a total of 97 ISSR markers ranging in size from 180 to >2000 bp, with 76.7% (±1.78) of the loci being polymorphic (S6 Table). The value obtained for θ^(II)^, i.e. an analogous parameter to Nei's Gst, was 0.080, thus being very close to the Bayesian estimate (Gst-B = 0.067), and 95% confidence intervals indicated that they are significantly higher than zero (Table 1), thus suggesting the existence of significant population differentiation. The average number of migrants (*N*_*e*_ *m*) estimated from this Gst-B value (3.48) indicated the existence of substantial gene flow among populations.

**Table 1:** Genetic diversity and population subdivision in *E*. *plorans* populations from the Segura River basin, provided by Hickory under the *f =* 0 model. 2.5% and 97.5% constitute the 95% confidence interval.

Parameter	Mean	SD	2.5%	97.5%
θ-I	0.278	0.025	0.230	0.332
θ-II	0.080	0.009	0.064	0.099
θ-III	0.055	0.004	0.047	0.064
hs[Claras]	0.220	0.006	0.208	0.232
hs[Socovos]	0.204	0.006	0.193	0.216
hs[Caravaca]	0.215	0.007	0.202	0.228
hs[Mundo*]	0.235	0.007	0.222	0.249
hs[Calasparra*]	0.213	0.005	0.203	0.223
Hs	0.217	0.003	0.211	0.224
Ht	0.233	0.003	0.226	0.240
Gst-B	0.067	0.006	0.056	0.079

The two populations carrying B chromosomes are indicated by an asterisk.

AMOVA showed the existence of a significant population structure, with 13.17% of total variance found among populations (P< 0.00001). Classifying the populations by B chromosome presence or absence, we found significant molecular variance between populations (F_ST_ = 0.15544) and between populations within groups (F_SC_ = 0.09292), but that found between B-carrying and B-lacking groups (F_CT_ = 0.06892) was only marginally significant (Table 2).

**Table 2:** AMOVA for ISSR markers in five populations of the grasshopper *E*. *plorans* collected in the Segura River Basin.

Source of variation	df	Sum of squares	Variance components		% Variation
Between groups	1	74.323	0.76016	Va	6.89
Between populations within groups	3	91.222	0.9542	Vb	8.65
Between populations	110	1024.646	9.31497	Vc	84.46
Total	114	1190.191	11.02933		
Fixation indices					
	F_CT_ =	0.06892			
	F_SC_ =	0.09292			
	F_ST_ =	0.15544			
Significance tests (10100 permutations)					
Va and F_CT_:	P-value = 0.09960±0.00272				
Vb and F_SC_:	P-value< 0.00001±0.00001				
Vc and F_ST_:	P-value< 0.00001±0.00001				

Populations were classified into two groups based on B chromosome presence or absence.

**Fig 1:**
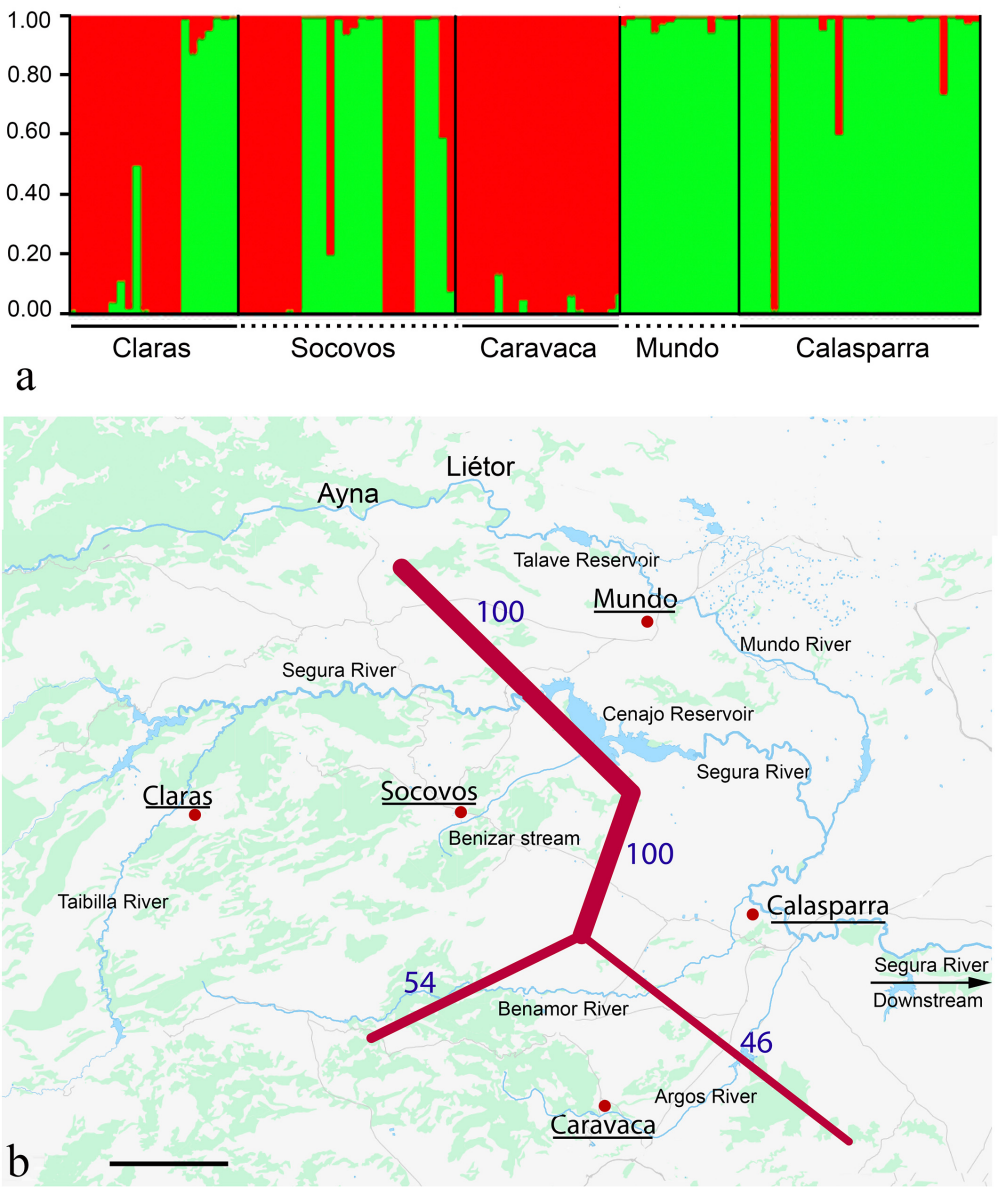
Barriers to gene flow between five natural populations of the grasshopper *Eyprepocnemis plorans* from the Segura River basin. (a) Ancestry of each individual to any of the two groups yielded by the Structure software, using ISSR markers. Note that most individuals from the B-lacking populations (Claras, Socovos and Caravaca) were included in Group 1 (represented in red color) whereas those from the two B-carrying populations (Mundo and Calasparra) were assigned to Group 2 (green color). (b) Map of the area analyzed showing the observed barriers to gene flow, detected by the Barrier software, and the location of the five populations analyzed, noted by a solid circle and their underlined name. Note that the strongest barrier to gene flow was found between the B-carrying population at Mundo and the B-lacking one at Socovos, placed at opposite sides of the Cenajo Reservoire. Scale bar = 10 Km.

The analysis with the Structure software revealed the existence of two genetically differentiated groups (K = 2) (S1 Fig), with individuals from the three B-lacking populations showing higher probability of assignation to group 1 (64.3% in Claras, 52.5% in Socovos and 93.9% in Caravaca) whereas most individuals from the two B-carrying populations were assigned to group 2 (98.3% in Mundo and 93.7% in Calasparra) (Fig 1A and S7 Table).

No IBD was found either using Pairwise-*Fst* (Mantel's test: r = 0.26, P = 0.29) or using Dice's dissimilarity index (r = 0.07, P = 0.56). In order to investigate possible causes of absence of IBD despite the significant population genetic differentiation found, we used Barrier v 2.2 to detect possible barriers to gene flow. It detected a very strong barrier between the populations carrying B chromosomes (Mundo and Calasparra) and the B-lacking ones (Caravaca, Claras and Socovos), with another medium-strength barrier between these latter populations (Fig 1B). This suggests the existence of geographical barriers as possible cause for the absence of IBD.

## Discussion

We present here an analysis of B chromosome distribution across a broad area, including estimations of gene flow between populations, which has revealed the existence of strong genetic barriers that coincide with geographical accidents impeding natural migration (canyons, inhospitable terrains, etc.). The most similar study is our analysis of the spread of the B_24_ chromosome around its place of origin (i.e. the Torrox population in the Spanish province of Málaga) [19] but, in that case, no genetic barrier impeded the spread of this new B variant towards east and west. In high contrast, the spread of the B_1_ chromosome from the coast to upstream populations in the Segura River basin has been interrupted by strong barriers to gene flow thus explaining the absence of B chromosomes in headwaters' populations.

Our present results support the hypothesis by Cabrero et al. [14] suggesting that populations lacking B chromosomes, located at about 600 m AMSL (e.g. Socovos in Benizar stream, Claras in Taibilla river and Caravaca in Argos river) (see Fig 1B), reached those localities prior to B chromosome arrival to the Iberian Peninsula, and these parasitic elements have not yet reached these high-altitude populations in their advance from the coast through later recolonizations. Our analysis of a B-specific sequence-characterized amplified region (SCAR) marker in populations from the Iberian Peninsula, Morocco, Greece and Armenia revealed scarce DNA sequence changes thus suggesting a recent origin for B chromosomes [18]. In fact, a same type of B chromosome (B_1_) is predominant in populations from the Iberian Peninsula, Balearic Islands, Morocco, Tunisia and Sicily, suggesting the recent spread of this B chromosome through Western Mediterranean areas [20]. Bearing also in mind that B_1_ is the most widespread variant in the Iberian Peninsula, on which basis it is considered the original B variant [6], we conclude that the arrival of B chromosomes to the Spanish Mediterranean coasts was a recent event, in consistency with the low *Fst* values found between the B-carrying and B-lacking populations analyzed here. This is reinforced by our repeated witnessing of B chromosome invasions in this species, and by the absence of B chromosomes in the headwater areas of the Segura River basin. In the Torrox (Málaga, Spain) population, the B_24_ chromosome completed its replacement of the B_2_ variant (from which it was presumably derived) in only a few years [21]. In the Mallorca Island, the B_1_ chromosome arrived first to the south-west area of the island, and then it spread towards the north, with B chromosome prevalence increasing one order of magnitude in the Pollença population in only ten years [22]. Recently, we have witnessed another B invasion in another Mediterranean river (Verde River) after monitoring the Otívar (Granada, Spain) population during a 35 year period (Camacho et al., submitted). This indicates that the pattern observed in the Segura River basin (B chromosome presence in the mouth but absence in the headwaters) is also valid for other Mediterranean rivers, and the repetition of such a pattern can only mean that B chromosomes arrived very recently to the Spanish coast. In this case, we can infer that the upstream B chromosome invasion from coastal to inland localities has not yet had time to reach the latter. In the case of the Segura River, the presence of strong barriers to gene flow, detected here, makes it even more difficult.

In a study of B chromosome frequency along the Segura River, from coastal populations up to Calasparra, Perfectti *et al*. [23] observed significant IBD for B chromosome frequency, in some years but not in others, being it especially apparent in the river mouth. It is remarkable that they found IBD in coastal populations plenty of cultivations where no geographical barriers exist for this grasshopper dispersion. These authors suggested high level of gene flow, recolonization, common descent and local adaptation as possible factors explaining the higher similarity between nearby populations, although they remarked the importance of metapopulation structure and recolonizations in the population dynamics of *E*. *plorans*. Our present results, however, have failed to show IBD for the ISSR markers in inland populations, and genetic differentiation among the analysed populations was low (*Fst* = 0.08). In the aphid *Schlechtendalia chinensis*, Ren *et al*. [24] observed high values of genetic differentiation among populations (*Fst* = 0.239) but no IBD, and suggested that this might be due to genetic drift or the low spatial scale of the studied populations. Likewise, the moth *Hyles tithymali* showed scarce genetic differentiation at several of the Canary Islands presumably due to fast and recent colonization [25], a fact that could also explain the low *Fst* values found in *E*. *plorans*.

The absence of B chromosomes from the head of the Segura River basin may be explained by the existence of strong barriers to gene flow found for the ISSR markers between the +B and-B areas. Topographical inspection demonstrates that the border between +B and-B localities coincides in each river with the presence of geographical accidents making a broad strip inhospitable for *E*. *plorans* grasshoppers. Most frequently, rivers pass through narrow canyons with arid soils surrounding them, with absence of irrigated fields and hence of herbaceous vegetation for food. For instance, in the Segura River, B chromosomes are present up to the Cenajo reservoir (see Fig 1B), which was built in one of these canyons [14]. The same is apparent in Segura's tributaries.

The barrier to gene flow between +B and-B populations explains the absence of B chromosomes above 600 m altitude in the Segura River and its Southern tributaries (those where Claras, Socovos and Caravaca samples were collected). In the Northern tributary (Mundo River), however, B chromosomes have reached the Liétor locality (600 m AMSL) [14], suggesting that the rugged terrain where the Talave reservoir was built, 6.5 Km downstream to Liétor (Fig 1B), did not impede the advance of B-carrying individuals. We cannot rule out that this geographical barrier might have been surpassed through accidental human transport.

Although the strong barriers to gene flow found in the Segura River basin explains the absence of B chromosomes at localities upstream the barrier, we cannot rule out the possibility that B chromosome presence is impeded, at some degree, by natural selection due to a low tolerance for B chromosomes at high altitude environments. A conceivable pathway could be associated with the cell-cycle lengthening effect that B chromosomes have in other species [26] causing a slowing-down in development [27–29] and thus a lengthening in life cycle. In high altitude locations, the available period for reproduction is shorter and this might be a disadvantage for B carrying individuals. However, the fact that B-carrying populations (e.g. Mundo, Hellín and Liétor, located by the Mundo River, the northern Segura's tributary) and B-lacking ones (e.g. Caravaca and Socovos, by southern tributaries), are located at similar altitudes (about 600 m AMSL) casts some doubts on this selective explanation and suggests that barriers to upstream colonization differ in strength from river to river.

## Materials and Methods

A total of 106 males and 25 females of the grasshopper *E*. *plorans* were collected at five natural populations in the Segura river basin, on the East of the Iberian Peninsula (see S1 Table). No specific permits were required for these collections. The locations sampled were not privately owned or protected in any way, and this field study did not involve endangered or protected species. To determine the number of B chromosomes per individual, males were anaesthetized and testes were dissected out, whereas females were injected with 0.1 ml of 0.05% colchicine in insect saline solution 6 h prior to anaesthesia and dissection of ovarioles. Testes and ovarioles were fixed in 3:1 ethanol:acetic acid and stored at 4°C. These materials were cytologically analysed by squash preparations in lactopropionic orcein and by the C-banding procedure reported in [30].

Body remains of each individual were frozen by immersion in liquid nitrogen and stored at -80°C. DNA was extracted with the kit GenElute Mammaliam Genomic DNA minipreps (SIGMA), following manufacturer's recommendations. DNA quantification was performed in a TBS-380 minifluorometer (Turner Biosystems) using Picogreen dye (Quant-iT PicoGreen dsDNA Kit; Molecular Probes, Invitrogen).

ISSRs markers were amplified by polymerase chain reaction (PCR) using the primers shown in S2 Table. PCR was carried out in a 25 μl reaction containing 1x PCR buffer, 240 μM dNTPs, 2 μM of primer, 1 U of Taq polymerase (New England, Biolabs) and 10ng of DNA. Reaction conditions included an initial denaturation at 94ºC for 3 min followed by 40 cycles at 94°C (40 s), 57°C (45 s) and 72°C (1.5 min), and a final extension at 72°C for 5 min. All PCR experiments were carried out in an Eppendorf Mastercycler ep Gradient and PCR products were separated in a 1.5% agarose gel with 1:1000 SYBR Safe (Invitrogen) and visualized with a gel documentation system (Gel Doc XR and ChemiDoc XRS, Biorad). The fragment sizes were determined using the molecular weight marker HyperLadder II (Bioline). The presence or absence of each fragment was coded as 1 and 0, respectively (see data file in S8 Table). As previously shown [17], ISSR markers show high repeatability in *E*. *plorans*. The analysis of ISSR allele frequencies was performed by Bayesian methods using the Hickory v1.1 software [31], under the *f = 0* model because *E*. *plorans* is a polygynandric organism [4] (see also [17] for model testing details), and specifying options for dominant markers. This provided population parameters on genetic variation and population differentiation.

In order to get a first approach on population structure, we performed an analysis of molecular variance (AMOVA) with the program Arlequin v3.5 [32] with 10,000 permutations and 20% maximum absence of data.

Population structure was also analysed with the Structure 2.3.1 software [33], which uses a Bayesian algorithm assuming an admixture model with K populations or groups characterized by a set of allele frequencies for each locus. Ten independent runs were carried out for each value of K (K from 1 to 6) and, for each run, 100,000 iterations were carried out after a burn-in period of 50,000 iterations. The best K was determined by the Evanno method [34] using the Structure Harvester website [35].

Pairwise-*Fst* values were calculated with the AFLP-surv software [36], and the possible existence of isolation by distance (IBD) was investigated by Mantel tests comparing the matrix of *Fst* values between each pair of populations, with the matrix of geographical distances between populations (S3 Table), both log_10_ transformed. All geographical distances were calculated as the crow flies. Mantel test was performed with the Zt-win software [37] with 100,000 random permutations. To minimize the problem caused by the dominant nature of ISSR markers, which implies assuming Hardy-Weinberg equilibrium to calculate allele frequencies, we also tested IBD by using Dice's dissimilarity index [38], instead of the pairwise-Fst values, for the Mantel test, by using the Famd v1.25 software [39] (see Dice's indices in S4 Table).

To ascertain whether the absence of IBD was associated with the existence of some genetic barriers to gene flow, we used Barrier 2.2 software [40], which connects the different populations by a Delaunay triangulation built from the geographical coordinates of each population. The barriers were then identified using Monmonier's maximum distance algorithm [40] from the matrix of genetic distances (Pairwise-Fst), obtained as explained above. The robustness of the results was estimated from 100 bootstrapped matrices.

## Supporting Information

S1 Fig Analysis of the best K by the Evanno et al. (2005) method.
(DOC)

S1 Table Number of individuals collected (N) and number of individuals analyzed for each ISSR marker. The two populations carrying B chromosomes are indicated by an asterisk.
(DOC)

S2 Table Primers used and number of markers obtained. (DOC)

S3 Table Matrix of Pairwise-*Fst* values (below diagonal) and geographical distance in Km (above diagonal) between the five populations analyzed. The two populations carrying B chromosomes are indicated by an asterisk.
(DOC)

S4 Table Matrix of Dice's dissimilarity index (below diagonal) and geographical distance in Km (above diagonal) between the five populations analyzed. The two populations carrying B chromosomes are indicated by an asterisk.
(DOC)

S5 Table Frequency of B chromosomes in the five populations analysed. The two populations carrying B chromosomes are indicated by an asterisk.
(DOC)

S6 Table Proportion of polymorphic loci in the populations analysed. The two populations carrying B chromosomes are indicated by an asterisk. SE = Standard error.
(DOC)

S7 Table Proportion of individuals assigned to every group in the Structure analysis. The two populations carrying B chromosomes are indicated by an asterisk.
(DOC)

S8 Table Data file of the ISSR markers analyzed in five natural populations of the grasshopper *Eyprepocnemis plorans*. (XLS)
