# Supplementary figures and images for: Geographical Barriers Impeded the Spread of a Parasitic Chromosome
Source: PLoS One. 2015 Jun 25;10(6):e0131277. doi: 10.1371/journal.pone.0131277 (PMC4482515; doi:10.1371/journal.pone.0131277)

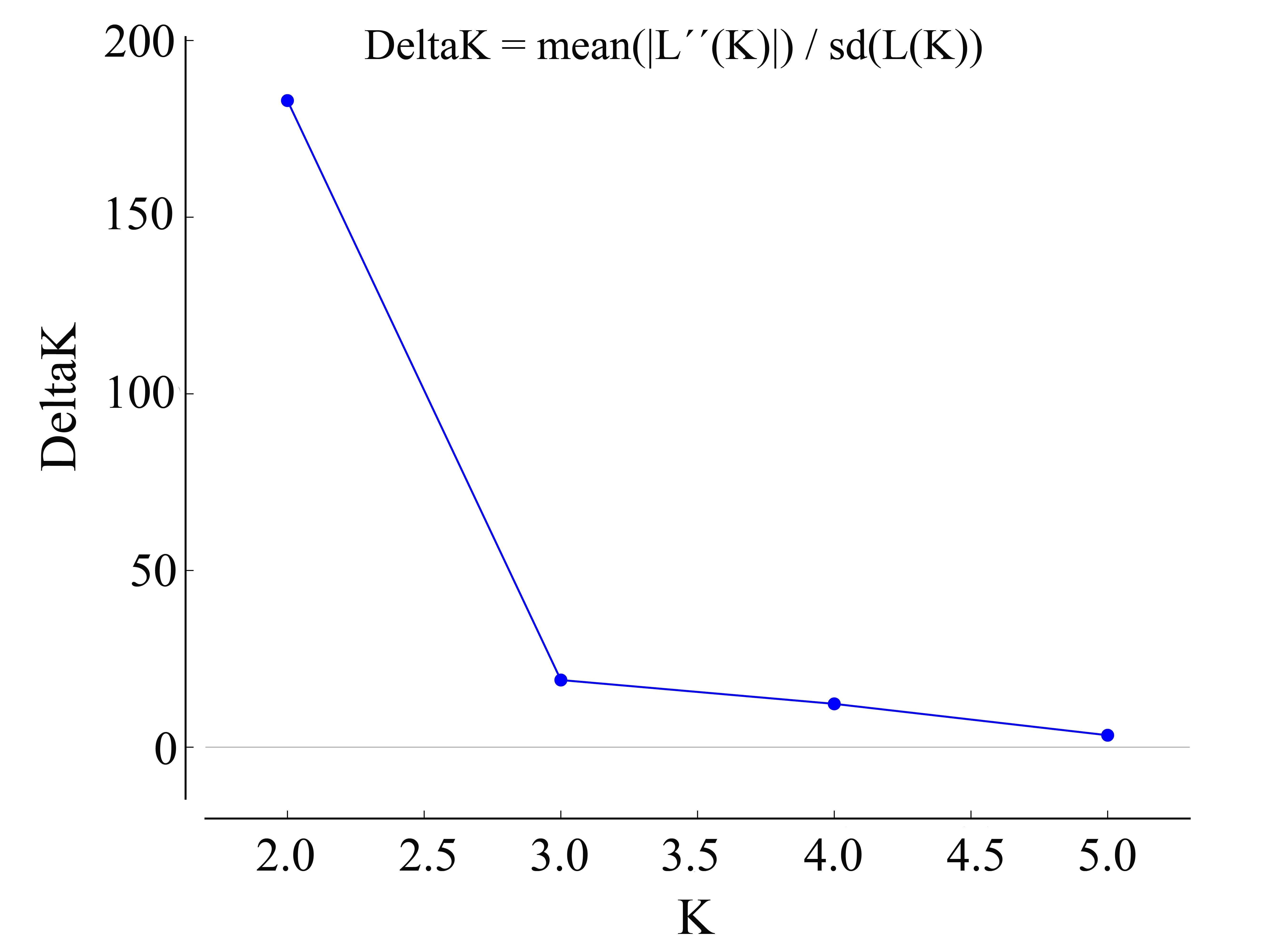


**S1 Figure. Analysis of the best K by the Evanno et al. (2005) method.**

Supplement: S1 Fig — (2005) method. (DOC) [file pone.0131277.s001.doc]
