# Supplementary material for: Geographical Barriers Impeded the Spread of a Parasitic Chromosome
Source: PLoS One. 2015 Jun 25;10(6):e0131277. doi: 10.1371/journal.pone.0131277 (PMC4482515; doi:10.1371/journal.pone.0131277)
Supplement: S1 Table — The two populations carrying B chromosomes are indicated by an asterisk. (DOC) [file pone.0131277.s002.doc]

| **S1 Table. Number of individuals collected (N) and number of individuals analyzed for each ISSR marker**. The two populations carrying B chromosomes are indicated by an asterisk. | | | | | | | | | | | | | | | | | | | | | |
| --- | --- | --- | --- | --- | --- | --- | --- | --- | --- | --- | --- | --- | --- | --- | --- | --- | --- | --- | --- | --- | --- |
|  |  |  |  |  |  |  |  |  |  |  |  |  |  |  |  |  |  |  |  |  |  |
| **Population** | **Province** | **Coordinates** | **Altitude** | **N** | **ISSR6** | |  | **ISSR7** | |  | **ISSR14** | |  | **ISSR26** | |  | **ISSR39** | |  | **ISSR43** | |
|  |  |  |  |  | **♂** | **♀** |  | **♂** | **♀** |  | **♂** | **♀** |  | **♂** | **♀** |  | **♂** | **♀** |  | **♂** | **♀** |
| Claras | Albacete | 38º 19.641’ N | 640m | 21 | 20 | - |  | 20 | - |  | 20 | - |  | 20 | - |  | 20 | - |  | 7 | - |
|  |  | 2º 13.091’ W |  |  |  |  |  |  |  |  |  |  |  |  |  |  |  |  |  |  |  |
| Socovos | Albacete | 38º 19.646’ N | 655m | 27 | 22 | 5 |  | 21 | 4 |  | 20 | 5 |  | 21 | 5 |  | 21 | 4 |  | 9 | 3 |
|  |  | 1º 58.064’ W |  |  |  |  |  |  |  |  |  |  |  |  |  |  |  |  |  |  |  |
| Caravaca | Murcia | 38º 6.501’ N | 570m | 23 | 13 | 10 |  | 12 | 10 |  | 12 | 10 |  | 13 | 9 |  | 11 | 9 |  | - | - |
|  |  | 1º 50.155’ W |  |  |  |  |  |  |  |  |  |  |  |  |  |  |  |  |  |  |  |
| Mundo* | Albacete | 38º 28.014’ N | 450m | 15 | 15 | - |  | 15 | - |  | 15 | - |  | 15 | - |  | 10 | - |  | 15 | - |
|  |  | 1º 47.398’ W |  |  |  |  |  |  |  |  |  |  |  |  |  |  |  |  |  |  |  |
| Calasparra* | Murcia | 38º 14.933’ N | 260m | 30 | 28 | - |  | 29 | - |  | 28 | - |  | 27 | - |  | 27 | - |  | 30 | - |
|  |  | 1º 41.672’ W |  |  |  |  |  |  |  |  |  |  |  |  |  |  |  |  |  |  |  |
| Total |  |  |  | 131 | 98 | 15 |  | 97 | 14 |  | 95 | 15 |  | 96 | 14 |  | 89 | 13 |  | 61 | 3 |
