# Supplementary material for: Geographical Barriers Impeded the Spread of a Parasitic Chromosome
Source: PLoS One. 2015 Jun 25;10(6):e0131277. doi: 10.1371/journal.pone.0131277 (PMC4482515; doi:10.1371/journal.pone.0131277)
Supplement: S2 Table — (DOC) [file pone.0131277.s003.doc]

| **S2 Table. Primers used and number of markers obtained.** | | |
| --- | --- | --- |
| **Primer** | **Oligonucleotide Sequence (5´->3´)** | **No. markers** |
| ISSR-6 | (CT)_8_-RG | 16 |
| ISSR-7 | (CTC)_4_-RC | 19 |
| ISSR-14 | (CT)_8_-RA | 11 |
| ISSR-26 | (CT)_8_-AC | 22 |
| ISSR-39 | (GA)_8_-YG | 18 |
| ISSR-43 | HVH-(TCC)_5_ | 11 |
| Total |  | 97 |
