# Supplementary material for: Geographical Barriers Impeded the Spread of a Parasitic Chromosome
Source: PLoS One. 2015 Jun 25;10(6):e0131277. doi: 10.1371/journal.pone.0131277 (PMC4482515; doi:10.1371/journal.pone.0131277)
Supplement: S3 Table — The two populations carrying B chromosomes are indicated by an asterisk. (DOC) [file pone.0131277.s004.doc]

| **S3 Table. Matrix of Pairwise-*Fst* values (below diagonal) and geographical distance in Km (above diagonal) between the five populations analyzed.** The two populations carrying B chromosomes are indicated by an asterisk. | | | | | |
| --- | --- | --- | --- | --- | --- |
|  | **Claras** | **Socovos** | **Caravaca** | **Mundo*** | **Calasparra*** |
| Claras |  | 21.9 | 41.4 | 40.5 | 25.5 |
| Socovos | 0.0410 |  | 26.9 | 21.9 | 25.4 |
| Caravaca | 0.0746 | 0.0418 |  | 40.0 | 20.0 |
| Mundo* | 0.0698 | 0.0641 | 0.1426 |  | 25.5 |
| Calasparra* | 0.0701 | 0.0555 | 0.1365 | 0.0381 |  |
