# Supplementary material for: Geographical Barriers Impeded the Spread of a Parasitic Chromosome
Source: PLoS One. 2015 Jun 25;10(6):e0131277. doi: 10.1371/journal.pone.0131277 (PMC4482515; doi:10.1371/journal.pone.0131277)
Supplement: S5 Table — The two populations carrying B chromosomes are indicated by an asterisk. (DOC) [file pone.0131277.s006.doc]

| **S5 Table. Frequency of B chromosomes in the five populations analysed.** The two populations carrying B chromosomes are indicated by an asterisk. | | | | | | | | | | | | | | |
| --- | --- | --- | --- | --- | --- | --- | --- | --- | --- | --- | --- | --- | --- | --- |
|  | **0B** | |  | **1B** | |  | **2B** | |  | **Total** | | |  |  |
| **Population** | **♂** | **♀** |  | **♂** | **♀** |  | **♂** | **♀** |  | **♂** | **♀** | **All** | **Prevalence** | **Mean** |
| Claras | 21 | 0 |  | 0 | 0 |  | 0 | 0 |  | 21 | 0 | 21 | 0 | 0 |
| Socovos | 22 | 5 |  | 0 | 0 |  | 0 | 0 |  | 22 | 5 | 27 | 0 | 0 |
| Caravaca | 13 | 10 |  | 0 | 0 |  | 0 | 0 |  | 13 | 10 | 23 | 0 | 0 |
| Mundo* | 10 | 0 |  | 6 | 0 |  | 0 | 0 |  | 16 | 0 | 16 | 0.375 | 0.375 |
| Calasparra* | 31 | 10 |  | 2 | 0 |  | 1 | 0 |  | 34 | 10 | 44 | 0.068 | 0.091 |
