# Supplementary material for: Geographical Barriers Impeded the Spread of a Parasitic Chromosome
Source: PLoS One. 2015 Jun 25;10(6):e0131277. doi: 10.1371/journal.pone.0131277 (PMC4482515; doi:10.1371/journal.pone.0131277)
Supplement: S6 Table — The two populations carrying B chromosomes are indicated by an asterisk. SE = Standard error. (DOC) [file pone.0131277.s007.doc]

| **S6 Table. Proportion of polymorphic loci in the populations analysed.** The two populations carrying B chromosomes are indicated by an asterisk. SE= Standard error. | | |
| --- | --- | --- |
| **Population** | **No. of polymorphic loci** | **% of polymorphic loci** |
| Claras | 76 | 78.4 |
| Socovos | 74 | 76 |
| Caravaca | 74 | 76 |
| Mundo* | 76 | 78 |
| Calasparra* | 72 | 74 |
| Average | 74.4 (SE= 1.67) | 76.5 (SE= 1.78) |
