# Supplementary material for: Geographical Barriers Impeded the Spread of a Parasitic Chromosome
Source: PLoS One. 2015 Jun 25;10(6):e0131277. doi: 10.1371/journal.pone.0131277 (PMC4482515; doi:10.1371/journal.pone.0131277)
Supplement: S7 Table — The two populations carrying B chromosomes are indicated by an asterisk. (DOC) [file pone.0131277.s008.doc]

| **S7 Table. Proportion of individuals assigned to every group in the Structure analysis.** The two populations carrying B chromosomes are indicated by an asterisk. | | | |
| --- | --- | --- | --- |
| **Population** | **N** | **Group 1** | **Group 2** |
| Claras | 21 | 0.643 | 0.357 |
| Socovos | 27 | 0.525 | 0.475 |
| Caravaca | 23 | 0.939 | 0.061 |
| Mundo* | 15 | 0.017 | 0.983 |
| Calasparra* | 30 | 0.063 | 0.937 |
